# Patients’ experiences with using virtual reality for comfort during source isolation: a qualitative feasibility study

**DOI:** 10.1080/1179271X.2026.2739003

**Published:** 2026-10-04

**Authors:** Tjitske Diederike Groenveld, Suzan Boekelder, Cariline Roosen, Harry van Goor, Getty Huisman-de Waal

**Affiliations:** ^a^ Department of Surgery, Radboud University Medical Center, Nijmegen, The Netherlands; ^b^ Department of Internal Medicine, Radboud University Medical Center, Nijmegen, The Netherlands; ^c^ Endoscopy Center, Radboud University Medical Center, Nijmegen, The Netherlands; ^d^ Scientific Center for Quality of Health (IQ Health), Radboud University Medical Center, Radboud Institute for Health Sciences, Nijmegen, The Netherlands

**Keywords:** Virtual reality, self-comfort, participation, source- isolation, nonpharmacological

## Abstract

**Background:**

Source-isolated patients endure feelings of loneliness, discomfort, and imprisonment with sensory deprivation and loss of autonomy. Supporting self-comforting strategies can increase comfort and autonomy and promote participation in recovery. We aim to explore whether and how source-isolated patients use virtual reality (VR) for self-comfort.

**Methods:**

A qualitative interview study, including a cohort of source-isolated patients. Patients received an VR intervention encompassing applications for relaxation and distraction and physical exercise during a maximum of 5 days. Patients were instructed using VR at least once a day for 20–30 minutes. Semi-structured face-to-face interviews were conducted after the intervention period or before discharge. Predefined topics were self-comforting strategies and patients overall experiences. Coding was guided by predefined topics, complemented with inductive coding of patients’ overall experiences.

**Results:**

Twenty patients were eligible, of whom two were excluded and eight declined participation. Ten patients, median (range) age of 56 (40–88) years, used VR for two to five days during a median isolation time of seven days. Beside the predefined self-comforting strategies two additional main topics emerged: “influencing factors on VR use” and “added value of VR”. VR was perceived to enhance positive thinking, mental distraction, active involvement, adaptation to limitations, and physical exercise. Soft- and hardware characteristics, access to the VR headset, patient characteristics, and ward environment may influence the perceived usability of VR. Perceived benefits of VR included promoting physical activity and providing an ‘escape’ from the hospital environment.

**Conclusion:**

VR has the potential to support patients’ comfort during hospitalization and may offer opportunities for greater autonomy by enabling patients to actively manage own physical and psychological comfort.

## Introduction

Around 18 percent of hospital days in medical-surgical and intensive care units involve source-isolation.^1^ Single-room isolation of patients with infectious disease or pathogen carriage is a well-established infection prevention measure to limit nosocomial transmission.^2^ Infrastructural measures, infection control protocols, and specialized nursing practices aim to minimize patient contact and prevent cross-contamination of pathogenic microorganisms. This includes, for example, the use of anterooms, personal protective equipment (PPE), and restrictions on clinician and visitor access. While some studies report positive elements such as cleanliness and the privacy of single rooms, most patients experience loneliness, discomfort and dissatisfaction with care, which negatively impacts psychological and physical comfort and may prolong recovery.^3–6^

Hospitalization itself already increases the risk of sleep disturbances, malnutrition, pain, and physical deconditioning due to illness and treatment effects.^7^^,^^8^ On top of this, source-isolated patients who are physically confined to one room endure feelings of imprisonment with sensory deprivation.^9^^,^^10^ This experience causes severe limitations in self-determination and autonomy, resulting in the feeling of little control over their well-being and recovery.^5^ In a situation where nurse workload is immense, the limited in-person caregiver-patient interactions are reduced to essential disease oriented activities. In this situation, and without (remote) alternatives for interaction, source-isolated patients are often compelled to regain control and autonomy themselves and rely on self-comforting strategies for their physical and psychological comfort.^2^^,^^6^^,^^11^

Comfort is defined as relief from physical discomfort and the experience of positivity and strength to cope with the challenges of illness, injury, and disability.^7^^,^^11^ It is considered a fundamental care need in the Fundamentals of Care Framework, representing the basis of nursing care and patient comfort.^7^^,^^12^ One way to foster comfort is through self-comforting strategies, defined as activities or actions individuals undertake to soothe themselves or alleviate symptoms^11^^,^^13^ Source-isolated patients often need to be encouraged to apply such self-comforting strategies and to autonomously care for their body, mind and spirit.^5^^,^^7^ Supporting self-comfort is important, as these strategies have been shown to increase comfort within healthcare settings and, in turn, to promote participation in recovery and health-seeking behavior.^11^ Different approaches can be used to support self-comfort. Traditional media such as radio and television provide sensory stimulation and an escape from isolation.^5^ Social media enables virtual interaction, however its use depends on patients skills, hardware and software possession, and the digital infrastructure of the hospital.^14^ More recently, intuitive patient-experience systems have been introduced on hospital wards, offering infotainment, education, home-like entertainment, and self-service options that enhance autonomy and comfort.^15^

Beyond traditional and social media, virtual reality (VR) provides immersive sensory stimulation and has been studied for various clinical applications.^16–20^ While often evaluated for anxiety and pain relief or for mobilization, qualitative studies also describe broader benefits for comfort, cognition, sleep, and providing an escape from the stressful or boring hospital environment.^17^^,^^18^^,^^21^^,^^22^ Its immersive nature and wide variety of virtual environments create opportunities for an individualized healing environment that can support patients physically, psychologically, and socially beyond the boundaries of a patient room.^11^^,^^23^ VR has not been investigated as a tool for self-comforting in patients isolated in single rooms. Evidence from other settings is promising. VR enhanced emotional comfort in hospice patients by providing joyful and calming applications that foster a sense of control and help them cope with illness and its symptoms.^24^ Similarly, a virtual ‘calm room’ improved awareness, calmness, and comfort in psychiatric inpatients.^25^ Furthermore, VR therapy has been shown to promote physical activity in patients rehabilitating at home.^26^ Reports of VR use for exercising within a hospital room are anecdotal but demonstrate feasibility in trauma and intensive care patients.^18^^,^^27^ VR appears particularly useful as a non-pharmacological self-managed tool for isolated patients. It is easy to operate, safe, and includes game-like applications that encourage repeated use, while also allowing for distant real-time support when needed. The head-mounted device requires little space at the bedside, charges with standard customer equipment, and can be disinfected by patients themselves after simple instructions to avoid high contamination levels.

Considering the diversity of VR applications that align with domains of self-comforting actions, the demonstrated usability and acceptability of VR hardware and software, and the relatively low costs (15–20 USD per patient per day), we conducted an qualitative feasibility study to explore the experiences of source-isolated patients using VR independently in a single room. Patients engaged in a set of mental and physical exercises designed to support their comfort. In this study, we applied an existing framework identifying self-comforting strategies attributed to the VR applications. Further, we evaluated usability and the overall experience of patients with regard to VR therapy.

## Methods

### Design

A semi-structured interview feasibility study was performed including a cohort of source-isolated patients with an infectious disease or carriers of antibiotic-resistant microorganisms admitted to a tertiary hospital.

### Theoretical framework

The topics of the interview guide reflected different self-comforting strategies extracted from the Calm framework: positive thinking, adaptation, working to get better, keeping busy and distracted (Appendix A).^11^ ‘Positive thinking’ describes the hope of patients that the situation improves and the thoughts about family or personal goals to help them through difficult times. ‘Adaptation’ refers to the patient’s ability to adjust and accept the situation in isolation. ‘Working to get better’ is about adjusting to and coping with symptoms in order to regain functioning. ‘Keeping busy and distracted’ refers to doing usual activities like watching television, pursuing hobbies, or other interests.^11^ Beyond these self-comforting strategies, the interviews also explored patients’ overall experiences with VR use.

### Study setting and recruitment

The study population consisted of a consecutive sample of source-isolated patients admitted to a single room of the general internal medicine ward of a university medical center in the Netherlands. Patients were verbally informed about the study within 48 hours of admission by a ward nurse. After obtaining informed consent, patients received a paper instruction on the self-management of the VR headset, supplemented by an individual verbal instruction and a hands-on demo of the VR headset and controllers, in the room. VR was prescribed at least once a day, with a duration of 20 to 30 minutes per session, and for five days in a row.^28^^,^^29^ This period accords with the average length of stay in isolation on the ward. Participants were free to perform more VR sessions per day at own discretion, judging their psychological and physical boundaries themselves. Participants were handed out a notebook to write down all types experiences immediately after each VR session and on each day to avoid recall bias.^30^

### Intervention

Patients first completed an introductory VR session during which the repository of applications was demonstrated and instructions were provided on how to navigate the exercises. After confirming that patients were able to use the intervention independently, the intervention period commenced. No information or instructions were given regarding self-comforting strategies or their potential benefits to prevent biasing their responses. Two different VR applications were offered. The application ‘SyncVR Relax & Distract’ (SyncVR Medical, Utrecht, The Netherlands) contains a wide range of relaxation games, videos, and exercises, each with a duration between 5 and 20 minutes. The application ‘SyncVR Fit’ (SyncVR Medical, Utrecht, The Netherlands) contains games that involve physical activity with a duration of approximately 10 minutes each and three levels in difficulty. The PICO Neo 3 Pro (Pico, Barcelona, Spain) was used as head-mounted VR device. The devices and applications were leased from SyncVR Medical (Utrecht, The Netherlands). SyncVR Medical was not involved in any way with study conduct, including design, analysis and manuscript preparation. No financial or non-financial relationships exist. All patients were provided with a head-mounted device for personal use, kept in their hospital room. Infection-control procedures were applied after use by each patient, following a standardized hygiene protocol (appendix B). VR use was deliberately not standardized with regard to session frequency, duration, timing, body position, or application choice. This pragmatic approach allowed patients to use VR according to their individual needs and preferences, reflecting the personalized nature of the intervention and enabling exploration of how patients naturally incorporated VR into their self-comforting strategies.

### Inclusion and exclusion criteria

Inclusion criteria were speaking and reading Dutch, age 18 years and above with no upper age limit, expected time of source isolation in a single room of two days or more. Exclusion criteria were dementia, an active delirium, vestibular abnormality, seizure disorder, migraine, audio-visual impairment, cognitive impairment and head injury.

### Data collection

Semi-structured face-to-face interviews were held in the single room at the end of the intervention period (5 days) or before discharge from source isolation by one researcher (SB), in absence of other patients, staff, or relatives. No repeat interviews were caried out. Interviews were audio-recorded. During the interviews, both the interviewer and the participant consulted the notebook for additional information (for the interview guide, see appendix A). The first interview was also used for appraising the topic list regarding self-comforting strategies, ‘influencing factors on the use of VR’ and ‘the added value of VR’. Demographic data were extracted from the electronic patient file.

### Data analysis

All interviews were transcribed ad verbatim and analyzed in Atlas.ti version 23 (Atlast.ti, Berlin, Germany). Thematic analysis was performed following the six-phase framework of Braun and Clarke’s.^31^ All transcripts were coded independently by two researcher (SB and CR). Although the interview guide was informed by the Calm framework, coding was conducted without predefined codes. Instead, all codes were generated inductively from the data. To minimize the risk of confirmatory bias and to prevent experiences outside the theoretical framework from being overlooked, coding was performed in two rounds. First, transcripts were coded while keeping the four self-comforting strategies in mind to identify experiences related to the framework. Second, transcripts were coded with the explicit question “What other experiences with VR emerge beyond these self-comforting strategies?” All emergent codes were subsequently grouped into themes and subthemes. A third independent researcher (TG) reviewed the coding and themes. The themes and subthemes were discussed until consensus was reached and final (sub)themes were defined. Differences between coders mainly concerned the interpretation and naming of codes or their allocation to themes and were resolved through discussion; when needed, a third researcher contributed to the final interpretation. During theme development, contrasting or deviant accounts were retained and used to refine the boundaries and interpretation of themes. Theme relevance was based on its contribution to the research question rather than frequency alone; however, the breadth of participant contribution was considered when interpreting findings supported by only a small number of accounts.

### Ethical considerations

The study was approved by the Medical Research Ethics Committee of Arnhem Nijmegen for non-Medical Research involving Human Subjects (No. 2023–16156). It was conducted according to the principles of the Declaration of Helsinki. The data were handled anonymously and securely stored in accordance with the European Union General Data Protection Regulation (EU GDPR) and the Dutch Medical Treatment Contract Acts (WGBO). Patients provided written informed consent for registration, use and publication of the research data.

### Rigor and reflexivity

The quality of the study was enhanced using the four concepts of Guba & Lincoln.^32^ All authors discussed the results and their implications, which enhanced credibility of the analysis. Member validation was not performed. However, during the interviews, the interviewer regularly paraphrased participants’ responses and invited them to confirm or clarify the interpretation, thereby reducing the risk of misinterpretation. The interviewer (SB) was trained to conduct qualitative research and had no prior experience with VR. Two VR therapy experts (TG and HvG) reviewed the interview guide for inclusiveness and appropriateness, enhancing dependability. Confirmability was supported by writing theoretical memos during the thematization process and reflecting on the first interview with the supervising researcher (GH). Although the interviewer also worked as a registered nurse on the ward during the study period, care was taken to ensure that no patient-caregiver relationship existed between the interviewer and any of the participants. Patients were informed about the occupation of the interviewer, no further information was provided to the patients. Involving two researchers (CR and TG) with experience related to VR practice fostered a higher level of conceptual thinking within the process of thematic analysis. Reliability was improved through written memos of the participants’ contexts and achieving intercoder agreement in the coding process. Finally, the study adhered to the consolidated criteria for reporting qualitative research (COREQ) for comprehensive reporting (appendix C).^33^

## Results

Twenty patients were deemed eligible for participation between February and June 2023. Ten patients (52%) were included (Table 1). Two patients were excluded because they were not source-isolated in a single room, eight patients declined for several reasons including fatigue, fear, lack of interest, feeling too ill, and overstimulation in the hospital environment. One patient, known to have fear of heights, was included but dropped out after the first (instruction) VR session due to unpleasant feelings. The patient was not interviewed but was considered regarding feasibility and safety of VR. Two patients reported adverse effects related to VR use. One experienced mild nausea during VR-based physical exercises and therefore primarily used relaxation videos. Another patient experienced the auditory stimuli as overwhelming.

**Table 1.** Characteristics of VR-use per participant.

Number of days	Total number of sessions	Time per session	Day period	VR apps
5	1	30 minutes	Night	Relax & Distract
5	5	10-15 minutes	Afternoon	Relax & Distract
5	5	≥ 30 minutes	^a^	Relax & Distract/Fit
4	2	≥ 30 minutes	Evening	^b^
3	3	15-30 minutes	Afternoon	Fit
3	9	20–30 minutes	Afternoon	Fit
2	2	20–30 minutes	Afternoon	Relax & Distract
5	2	10 minutes	Night	Relax & Distract
5	15	30 minutes	Afternoon	Relax & Distract/Fit
5	15	15 minutes	^a^	Relax & Distract/Fit

^a^ Various moments of the day. ^b^No information available.

The median age of the ten evaluated patients (6 female, 4 male) was 56 years, ranging from 40 to 88 years. Five patients were isolated due to an infectious disease (diarrhea, coronavirus, respiratory virus, herpes zoster), while the other five were isolated because of suspected carriership of antibiotic-resistant microorganisms (MDRO or MRSA)(appendix D). Reasons for hospitalization and comorbidities varied widely, including dermatologic conditions as well as diverse infectious and internal diseases. Median duration of isolation was seven days, ranging from 4 to 13 days.

Seven participants daily utilized VR. The number of sessions on a day varied between 1 and 3. Session duration was between 10 minutes and 30 minutes or occasionally more. Eight participants predominantly exercised in bed, one while sitting in a chair and one while standing. Only 3 participants noted comments in the provided notebook, which mostly regarded technical aspects of the VR applications.

### Interview findings

The duration of the interviews varied between 15 and 45 minutes, with a mean duration of 30 minutes. Appraisal of the first interview did not result in changes of the topic list or the structure of the following interviews. Findings were categorized into three main topics: ‘self-comforting strategies’, ‘influencing factors on the use of VR therapy’, and ‘the added value of VR therapy’. The first topic was derived from the interview guide, while the latter two emerged from thematic analysis of patients’ overall experiences with VR. Within each topic themes and subthemes were defined based on thematic analysis (Appendix E).

#### Self-comforting strategies

‘Positive thinking’, ‘keeping busy and distracted, ‘adaptation’, ‘working to get better’, and ‘influence on physical condition’ were found as main themes.

##### Positive thinking

Patients perceived VR to support mental positivity, allowing them to momentarily stop worrying and realize that they could not change their situation at that moment. Feeling restricted by being physically confined to their room, the virtual world enabled them to imagine themselves outside the room. As a result, they felt more present and less stressed about the uncertainty of their situation. One patient compared VR with a religious experience, stating that it had a positive effect on his spirit.

I: How does it [VR] positively influence you?

P: On the spirit. When you are worried about something, then by looking at something like this, everything feels more manageable. […] I am a very religious person: I meditate, I go to church every Sunday and so on. And what do you experience there? It is hard to put into words. And with this headset, something similar happens, you enter another world that is difficult to describe.” (male, 76 years)

##### Keeping busy and distracted

This self-comforting strategy focusses on passing time and distracting the mind. Patients indicated that both are necessary during hospitalization. Patients perceived VR as helping to distract their mind and to pass time more easily. ‘Staying occupied’ and ‘relaxation’ were identified as important subthemes.

###### Staying occupied

Patients perceived VR as helping to stay occupied during their admission. The headset, its possibilities, and the virtual world aroused their curiosity. Most patients had not previously used a VR headset, which enhanced their experience. This encouraged them to explore a variety of content including games and movies, contributing to a sense of time compression during their hospital stay.

Well, time just goes by faster. When you take off the headset after about half an hour, that half hour has already passed, but it doesn’t feel like you’ve been at it for that long. (male, 62 years)

Yes, well, it helps just for those moments when you think, I would actually like to have contact with someone or do something, then you can take the headset and you can actually ‘sort of’ do something. (male, 62 years)

Interestingly, some patients perceived their hospitalization as a busy period, characterized by physical care, visits from health personnel and family members, and various examinations. Consequently, they sometimes encountered difficulties in finding the time and energy to engage in VR throughout the day. This was one of the reasons why patients used VR only once daily. Other reasons included low familiarity with VR, limited digital literacy, and feeling to ill.

###### Relaxation

Patients perceived VR use to facilitate relaxation and offer distraction. Patients felt fully immersed in the virtual world during relaxing videos, and felt enabled to unwind both their thoughts and bodies.

Yes, it did calm me down. Yes, you are distracted right away. […] So it does give peace of mind. It definitely does. (female, 50 years)

Some patients found the process of starting the VR headset and navigating the software to be time-consuming. They experienced initial frustration, which hindered their ability to relax. However, most were able to regain focus and enjoy the VR therapy after the start-up phase.

##### Adaptation

Some patients experienced difficulty to adapt to the restricted environment of their hospital room. Some of these patients felt VR supported them in adjusting to this setting. Two subthemes were identified, being ‘personal interaction’ and ‘autonomy’.

###### Personal interaction

Patients experienced reduced contact with their social networks due to isolation or being bedbound. Additionally, every person entering the room was dressed in accordance with isolation precautions, changing interaction. VR was used during moments of solitude.

Not everyone just walks into the room. The people who do come are from your team [ie clinicians]. That’s purely for medical reasons. My wife does visit regularly, but other than that I don’t have anyone coming by often, so yes, it can feel quite lonely. (male, 62 years)

###### Autonomy

Patients appreciated personal ownership of the VR headset along with their ability to choose content and control duration of use. They reported this helped them feel more like themselves instead of being defined by their illness. Patients felt it shifted their attention toward their abilities during isolation especially when performing exercises. This was valued important, as patients reported feeling healthier by engaging in activities that differed from their usual hospital routines. Doing active games, meditate, or experiencing nature helped restore a sense of their everyday reality.

[…] and also more of a way to get away from the reality of this room, you know. So you are still between these walls, which also makes you behave a little bit as if you are confined to those four walls, and the headset gave me, at least, a way back to my own reality. […] So I felt much less at the mercy of being a patient, and just a bit more myself so to speak. Less in the role of the patient lying in bed and being handed everything […]. (male, 55 years)

##### Working to get better

Although patients used VR for self-comfort, they did not associate this comfort with their physical recovery. Consequently, they did not purposely use VR therapy for this purpose. Patients primarily relied on medication for their recovery.

I: How did VR therapy affect your mental state in isolation?

P: Yes, I can’t really say much about that because I had COVID, […] Later on you can talk more about the impact on your mental state, but right now you still have to endure COVID, and I don’t think an amazing headset changes anything about that. (male, 76 years)

I: Did you feel it [VR therapy] had an effect on your recovery?

P: Hmm, I’m not sure. […] I think it is more like you have this nasty bug and it just has to be defeated. How you feel during that might help a little but it’s really only a very small effect. It really has to be the antibiotics doing the work that solves things. But I can imagine if it is an illness where it’s important to move, then it could have more of an effect. […] Like when you have to rehabilitate from something. (female, 40 years)

##### Influence on physical condition

VR may have a positive impact on physical fitness, sleep, and physical discomfort. Patients indicated this as an important advantage over the other devices such as TV or smartphone.

###### Impact on physical fitness

Some patients in this study used VR as a tool to increase exercise within the patient room. It was considered the only available way to be physically activate and offered greater variety in mobility exercises.

The activity, for me, is another topic. You can relax with the television or radio or something else. But there’s no other way to move around in the room when, like me, you have to stay there for three, four, five days. I am not allowed to use the exercise bike [in the hallway] or go for a walk there, so yes, then this is really good. (male, 59 years)

As a result, patients reported both physical and mental benefits during isolation. Patients reported VR exercises to be more enjoyable and engaging compared to conventional physiotherapy, enhancing feelings of commitment and stamina.

It actually did, in a small way, what boxing normally does for me. […] I felt fitter and stronger, also mentally […] It is more positive than when I didn’t have the VR headset and was just lying here. […] Somehow, with that headset, you get activated to actually do it, you know. It helps you overcome a barrier that you might normally have. (male, 55 years)

###### Impact on sleep

Most patients used VR in the evening or at night, when it was quieter and there were fewer interruptions from healthcare staff. Patients reported VR to provide distraction and movement, which helped patients fall asleep. Nature videos were especially popular during nighttime due to their relaxing effect.

###### Impact on physical discomfort

Patient experienced that immersion in a captivating virtual world provided relief from symptoms causing physical discomfort. However, VR therapy was considered complementary and not a standalone treatment in this context.

Well, while you’re engaged with it [VR], the pain does fade a bit into the background. But it wasn’t the solution. It does need to be combined with painkillers. In combination with the painkillers, it works faster. (female, 51 years)

#### Influencing factors on the use of VR

Patients reported that software and hardware factors, the hospital environment, and patient characteristics influence the use of VR therapy, both positively and negatively.

##### Soft- and hardware

The usability was of great importance to patients. The VR-software was generally considered easy and intuitive. Most participants considered the variety of games and movies adequate. However, they were critical about the content and layout of the virtual world.

Look, if it were even more realistic, I could relax even more. That you can really, despite being in isolation, feel as if you are in the outside world. (male, 54 years)

Some patients found the weight of the headset limiting to its use. Using the controllers was sometimes experienced as physically challenging or distracting from the content.

##### Environmental factors

Environmental factors, including access to the VR headset and ward characteristics, should be accounted for to enable optimal VR use.

###### Independent access to the VR headset

Patients reported a preference for flexible, self-directed use over a prescribed program and expressed that keeping the VR headset in their room was essential to enable independent use. Bedbound patients particularly valued having the headset readily accessible within direct reach. Additionally, the ability to choose content was considered necessary to support patient autonomy and enable personalization with some patients expressing specific preferences for more options.

I thought that [having the VR in the room] was a real gift, and I liked that I could use it on my own, fitting it to my own schedule above all. Time is limited here, you wouldn’t think that about being in a hospital, but it’s true. (male, 55 years)

###### Ward characteristics

Patients reported hospital equipment, such as bed rails and IV systems, to occasionally restrict arm movement and interfere with VR use. Patients indicated that having a chair and sufficient space to stand or move supported them in adopting their preferred position for VR exercises.

Some participants reported that not all nurses had the knowledge to support VR use when needed, which limited patients’ engagement with the VR.

##### Patient characteristics

Expectations, physical characteristics, and digital familiarity may impact patients’ ability to use VR and comfort themselves with it.

###### Expectations

Patients had varying expectations of VR. Most anticipated it to be fun and distracting, while some expected it to be more suitable for young children and saw no added value compared to other media. In the end, most patients viewed VR as a positive experience though some expected higher quality content, for example the videos.

I thought, well, I have two favorite TV programs which help me relax, two programs every day. And, I thought, maybe the VR headset could give me the same feeling. (female, 57 years)

###### Physical characteristics

Pre-existing nausea, back pain, severe illness, and limited physical condition or motivation were reported as barriers to VR use. Although nausea is a known side effect of VR, patients did not experience it nor did pre-existing nausea worsen.

[When asked about the impact of feeling unwell on using the VR] Well, I wasn’t planning to use it. Just lying here is already enough. Even the TV, I sometimes turn it on, but it’s just on and I’m not really interested in watching it. I’m not really interested in anything. I just want to rest. (female, 50 years)

###### Digital familiarity

Patients who considered themselves digitally skilled reported needing less instruction than those who felt digitally inexperienced. Prior experiences with technology appeared to positively influence the ability to use VR. Notably, the oldest patient (88 years) expressed confidence in mastering the device with nurse’s support in handling both software and hardware.

##### Added value of VR

VR was perceived as most valuable for engaging patients in interactive exercises that involved movement. The immersive nature of VR was perceived to distinguish VR from other forms of media, offering patients an escape from the hospital environment into a relaxing virtual world. Additionally, the ability to store more content on the VR headset was considered beneficial.

However, some patients found VR less accessible than radio or television due to the time required to set it up and ensure it worked properly. They felt that watching a movie on television could provide the same relaxing effect as VR.

I think that if you just want to help someone relax, you might as well put on the television set and let them watch a program. That was my idea. (female, 88 years)

When you have the possibility to also move in bed and, yes, stretch a bit more. That is also good. For me, that was the most valuable aspect of the use. (male, 59 years)

## Discussion

Patients perceived self-administered VR as supporting several mental, physical, and behavioral aspects of comfort during single-room isolation. Reported benefits include positive thinking, mental distraction, active involvement, adaptation to boundaries, and physical exercise. Patients also identified soft- and hardware characteristics, access to the VR headset, patient characteristics, and ward environment as factors influencing the usability of VR.

Patients received general information about the use of VR for relaxation, distraction, and physical exercise. They were not informed about its potential role as a self-comforting strategy that could positively influence recovery, nor did they receive prior education or guidance in self-comforting strategies. This may partly explain why patients generally did not associate VR use with recovery and primarily relied on pharmacological treatment. These finding may also reflect a broader lack of awareness among patients of their own potential in supporting recovery through self-comforting strategies. Future implementation and evaluation of VR for comfort may therefore benefit from providing patients with education on self-comforting strategies and on how enhancing their own physical and psychological comfort may contribute to recovery. Nonetheless, patients did report effects attributable to VR that aligned with self-comforting strategies. However, the interview structure, focused on self-comforting strategies, may have reinforced this association. Several VR applications, including relaxation exercises, immersive nature videos and motivating physical exercises, may have prompted the subconscious use of these strategies. An explanatory mechanism could be the triggering of patients’ sense of autonomy by redirecting attention from limitations to abilities.

Due to the exploratory nature of this study, VR was not directly compared to other self-comfort tools, such as watching TV, video calls with family, or listening to music. However, patients spontaneously mentioned in interviews they perceived added value of VR over other media, like TV. This was attributed to the immersive and interactive qualities of VR, particularly in the context of physical exercises. Patients perceived immersive VR applications as offering a welcome escape from the ‘nocebic’ hospital environment, reflected in their descriptions of time compression, virtual travel outside the room, and distraction from negative thoughts.^23^ Patients also perceived VR as lowering the threshold for engaging in physical exercise by providing a wide variety of interactive content, although most used VR while lying in bed. While it was not directly compared to standard mobilization tools such as handgrips, bed-steppers, or bike trainers, VR was appreciated for its easy access and usability in supine and seated positions. Immobilization during hospitalization, particularly among critically ill patients, is a well-known challenge as it negatively affects recovery and post-discharge quality of live.^34^ Clinicians often depend on patient engagement to promote mobility. VR can support this process by encouraging self-initiated activity. Due to the qualitative nature of this interview study, effects on mental and physical recovery were not measured. However, VR-based exercise therapy has demonstrated positive effects across various conditions, largely driven by the enjoyment of exercising and high treatment adherence.^35^

Usability was perceived differently by patients and could be related to three aspects eg technology, patient factors and room environment. Some patients found VR less accessible compared to traditional media such as radio or television. This seemed not only to be related to the multiple steps required to operate the headset, controllers, and to navigate the menus, but also to patient’s illness. Furthermore, the bedside environment such as bedrails, intravenous lines connected to bedside pumps, and limited space around the bed, was perceived as limiting movement and ease of use. These findings highlight the importance of user-friendly design and implementation because ease of use is known to influence both the uptake and perceived added value of VR therapy.^36^^,^^37^ One could even think of interior design adaptations to facilitate VR exercising such as form and position of furniture and equipment.

Patient reported inadequate support from nurses in handling the VR device, busy schedules, and unannounced daytime interruptions, often forced them to move VR session to quieter evening hours. Similar issues were reported by patients with blunt thoracic trauma on a surgical ward.^18^ These limitations on patient’s self-efficacy to administer VR illustrate the restricted uptake by clinicians of VR as a therapy and as a self-comforting tool that contributes to recovery in hospital. This is not unique to VR for comfort, but reflects the broader underestimation by clinicians and hospital organizations of the value of non-pharmacological interventions and physical spaces in promoting patient comfort and early recovery on general wards.^34^^,^^38^ Given the constraints of the physical environment and particularly of an isolation room, VR has the potential to provide a wide variety of digital healing environments.

Patients were offered a menu of VR applications targeting mental, cognitive, and physical health, with multiple exercises within each category. Despite this variety, patients emphasized the importance of personalized and adaptable VR content to enhance usability. Achieving on-demand software adaptability and personalization remains challenging due to the wide variation in biopsychosocial characteristics, including differing expectations, susceptibility to immersion and presence in VR, interaction preferences, and levels of digital literacy of patients.^16^^,^^39^^,^^40^ Emerging software developments, such as no-code platforms for creating interactive VR applications, already applied in medical training, may facilitate individual adaptation in clinical settings.^41^

### Strengths and limitations

An important strength of this study is its pragmatic design, allowing patients to use VR therapy at their own discretion, giving them control over timing, duration, and type of content (relaxation vs physical exercise). This approach offers new insights into patients’ comfort needs, the extent to which they spontaneously or subconsciously employ self-comforting strategies and how they independently engage with digital tools like VR to support these strategies.

This exploratory study comprised a small study population, limiting the transferability of the findings and the likelihood that all relevant experiences were captured. Although data saturation was not fully achieved, as some topics were mentioned only once, the level of thematic convergence was considered sufficient for the exploratory aim of understanding how patients perceived VR as supporting self-comforting strategies. Participants were source-isolated for different indications, introducing some clinical heterogeneity. However, all were hospitalized with an acute illness and experienced comparable isolation restrictions and PPE requirements. Although differences in experiences related to the underlying indication for isolation cannot be excluded, these shared circumstances may have resulted in similar experiences of isolation and VR use.

Patients focused more on the feasibility aspects of VR hard- en software than on its perceived role in supporting comfort or facilitating self-comforting strategies. This may have been influenced by the absence of prior patient education about self-comforting strategies and the choice not to prime patients on this specific aim. As a result, the range of topics discussed and the depth of reflection on VR as a comfort intervention may have been limited. Furthermore, not explicitly introducing VR as a self-administered tool for comfort may also have influenced participants’ perceptions of its usability. At the same time, the findings provide insight into how patients naturally engaged with VR for comfort, while highlighting opportunities where additional education and instruction may enhance its therapeutic value. This pragmatic approach also informed our choice of a semi-structured interview guide, enabling us to explore whether and how patients used VR for self-comfort. Although the theory-informed interview guide may have introduced a risk of confirmatory bias, we sought to minimize this by using open-ended questions, incorporating disconfirming prompts during interviews, applying inductive coding, involving multiple coders, and conducting per debriefing.

Another potential source of bias was the timing of the interviews, which varied between immediately after the 5-day intervention period and before discharge or the end of isolation. These differences, resulting from practical considerations, may have introduced variability in recall. To reduce recall bias, participants were provided with a diary to record their experiences during the intervention. However, only a few participants used the diary, mainly to report technical issues, limiting its intended contribution. Despite these limitations, the interview data were considered sufficient to address the exploratory aims of this study.

The qualitative design of this study did not allow for the measurement of VR’s effect on the comfort of source-isolated patients. In retrospect, it would have been feasible to unobtrusively collect continuous health data from wearable continuous monitoring devices and examine temporal associations with VR use.^42^ Additionally, no quantitative data were collected on usability issues, such as technical failures or assistance needs. Consequently, these aspects could not be reported systematically. However, participants’ interview accounts provided valuable insights into the nature and perceived impact of these issues from the end-user perspective.

A further limitation is that patients who declined participation often reported fatigue, feeling too ill, overstimulation, or lack of interest. As reasons for non-participation were not systematically explored, it remains unclear whether these were related to VR itself or to study participation. Consequently, the findings may overestimate the perceived feasibility and acceptability of VR. Future feasibility studies should further explore reasons for non-participation to better understand barriers to VR use and acceptability. At the same time, VR is unlikely to be suitable for all hospitalized patients and should be considered one of several non-pharmacological interventions that may support patient comfort, rather than a universal solution.

### Clinical and research impact

VR has the potential to provide patients with a means to support their own comfort during hospitalization, thereby fostering autonomy. Since comfort is considered a core element of nursing care, VR could also serve as a supportive tool for nurses in fulfilling this role. This concerns in particular settings where direct caregiver-patient interaction is limited and workloads are high.^7^^,^^12^ VR is a safe and accessible non-pharmacological intervention that can be prescribed by nurses for promoting comfort, relaxation, sleep, and mobilization, strengthening nurse autonomy in clinical practice. At the same time, VR may encourage patients to move beyond a passive role as a recipient of care and to take an active role in enhancing their own comfort .^43^^,^^44^ This requires a shift in habits for both nurses and patients but holds promise for a more balanced and participatory care dynamic.^43^

From a research perspective, future studies should assess the clinical impact of VR in comparison with existing non-pharmacological comfort interventions such as television, music, radio, or bedside exercise tools. A critical prerequisite, however, is the seamless integration of VR therapy into clinical practice before study initiation, since inadequate integration may interfere with contextual factors and distort treatment effects.^18^ Active involvement of nurses in this process is essential, as their full engagement has been shown to enhance the uptake of new technologies.^45^^,^^46^ Importantly, all stakeholders should acknowledge that the implementation of VR initially requires an investment of time and effort, but may turn into greater autonomy, improved comfort, enhanced quality of care, and time-savings.^47^ Future research could expand beyond patients in isolation exploring whether VR may facilitate the use of self-comforting strategies in broader inpatient populations. Another promising direction lies in social virtual reality, which enables virtual social interactions and allows individuals to connect regardless of geographic or mobility limitations.^48^

## Conclusion

VR was perceived by patients to stimulate positive thinking, keeping busy and distracted, adapting to confinement in isolation, and physical exercise, and may provide distraction from the stressful experience of a hospital admission. Characteristics of the soft- and hardware, hospital (room) environment and patients themselves may influence the perceived usability of VR therapy.

## Supplementary Material

Appendix B Hygiene protocol PICO Head Mounted Display.pdf

Appendix A Interview Guide.pdf

## Data Availability

The datasets used and/or analyzed during the current study are available from the corresponding author on reasonable request.

## Transparency

### Declaration of financial/other relationships

The authors have no relevant affiliations or financial involvement with any organization or entity with a financial interest in or financial conflict with the subject matter or materials discussed in the manuscript. This includes employment, consultancies, honoraria, stock ownership or options, expert testimony, grants or patents received or pending, or royalties. Peer reviewers on this manuscript have no relevant financial or other relationships to disclose.
