# Supplementary material for: Patients’ experiences with using virtual reality for comfort during source isolation: a qualitative feasibility study
Source: Patient Relat Outcome Meas. 2026 Oct 4;17(1):123–35. doi: 10.1080/1179271X.2026.2739003 (PMC13644613; doi:10.1080/1179271X.2026.2739003)
Supplement: Supplementary file 1 [file DPRO_A_2739003_SM3760.pdf]

Appendix A. Interview guide

<u>Introduction</u>

- Introducing researcher
- Illustrate aim of study
- Emphasize the importance of hearing the ideas and experiences of interviewee
- Give an indication of interview duration: 30 to 45 minutes
- Ask permission to start audio recorder and protection of personal data

**Topic**	**Main question**	**Sub questions**
General experience	You used the virtual reality headset during your period in source-isolation. What was your experience with the use of the virtual reality headset?<br>(use input diary)	For how long did you use the virtual reality headset at a time? (use input diary)<br>What did you think about this?<br>How did you experience your time in source-isolation?
Reason for use	When did you use the virtual reality headset during your period in source-isolation?<br>(use input diary)	There were two programmes from which you could choose. Which programme or element did you mostly use?<br>Why did you choose for this programme or element?<br>*Order of the next topics depends on the answers of the participant*
Positive thinking<br>Keeping busy<br>Distraction<br>(SyncVR Relax & Distract)	What impact did the virtual reality headset have on your mental state during source-isolation?<br>What was the influence of the virtual reality headset on distraction during your stay in isolation?	What did this do to you?<br>Ask further questions about items raised by the participant<br>*Psychological elements:*<br>*- loneliness*<br>*- gloom/dreariness*

		- *fear*<br>- *insomnia*
Working to get better<br>Adaptation<br>(SyncVR Fit)	How did the virtual reality headset affect your activity/movement pattern during source-isolation?<br><br>How did the virtual reality headset affect your daily routine while source-isolated?	How did this affect your independence?<br><br>Ask further questions about items raised by the participant<br><br>*Physical elements:*<br>- *physical limitation/movement*<br>- *sensory deprivation*<br>- *interruption of normal routines, such as day/night rhythm*
Conclusion	What effect did using the virtual reality headset have on your recovery?<br><br>How could the use of the virtual reality headset on a nursing ward be improved?<br>→ targeted use<br>→ importance of technological knowledge	What would you recommend to other patients about using the virtual reality headset in isolation?<br><br>What are your ideas about using the Virtual Reality headset for patients who are not in isolation?
