# Supplementary material for: Patients’ experiences with using virtual reality for comfort during source isolation: a qualitative feasibility study
Source: Patient Relat Outcome Meas. 2026 Oct 4;17(1):123–35. doi: 10.1080/1179271X.2026.2739003 (PMC13644613; doi:10.1080/1179271X.2026.2739003)
Supplement: Supplementary file 2 [file DPRO_A_2739003_SM3761.pdf]

17-12-2021 versie 1.1

HYGIENE PROTOCOL PICO Head Mounted Display (VR headset)

Disinfection PICO Head Mounted Display (VR headset)

Reusable, non-disposable components

- VR headset (goggles)
- Headband (consisting of 2 components)
- Charger
- Controller
- Lidded storage box

Disposable components

- User manual Virtual Reality headset
- Application catalogue

General remarks:

1. All reusable components must be used on a patient-specific basis.
2. The VR headset is available for use on every care units.
3. The VR headset is allowed to be used by patients who are source-isolated.
4. Disinfectants to be used: OxyFoam in combination with a disposable microfiber cloth, Bacillol 30 wipes or Oxy wipes S and a UV box (D25).

Measures for cleaning and disinfecting during use:

1. Instruction for patients: hands need to be disinfected before using the Virtual Reality headset.
2. The Virtual Reality headset needs to be stored in a lidded box suitable for cleaning and disinfecting (for example plastic box) without fabrics. This box is patient bound and needs to be cleaned and disinfected as well after every patient.
3. The user manual and application catalogue of the Virtual Reality headset for patients needs to be printed and replaced after every patient.

Measures for cleaning and disinfecting after use:

A cleaning and disinfecting protocol applies to the reusable components:

1. Disinfect your hands first.
2. Set up an organized system for separating clean and dirty components.
3. All reusable components are fully cleaned and disinfected after each patient.
   Be aware of also cleaning and disinfecting the riffles of the headset and areas affected by shadowing (areas missed within the UV box).
4. In case visible contamination is present (ultrasound gel, blood, body fluids, etc.): first remove the contamination with a disinfection wipe, then clean and disinfect again using a fresh disinfection wipe.
   In case of non-visible contamination, a single action suffices; cleaning and disinfecting are performed in a single step using one disinfection wipe.
   For every component a new disinfection wipe should be used: headset, controller, charger
   - The headset needs to be fully dismantled for cleaning/disinfection
     - detach the small headband from the big headband (Pico neo 3 pro en Pico G2)

- Detach headband from the headset (when using Pico G2)
    - detach headset from charger (if necessary)
    - detach rings around the lenses (when using Pico G2)
    - fully lengthen the body of the headset using the focus button (when using Pico neo 3 pro)

<u>5. Disinfection of every component:</u>

- VR headset (goggles)
- Headband
- Charger
- Controller
- Lidded storage box

6. Then the headset, controller(s) and charger need to disinfected using the UV box.

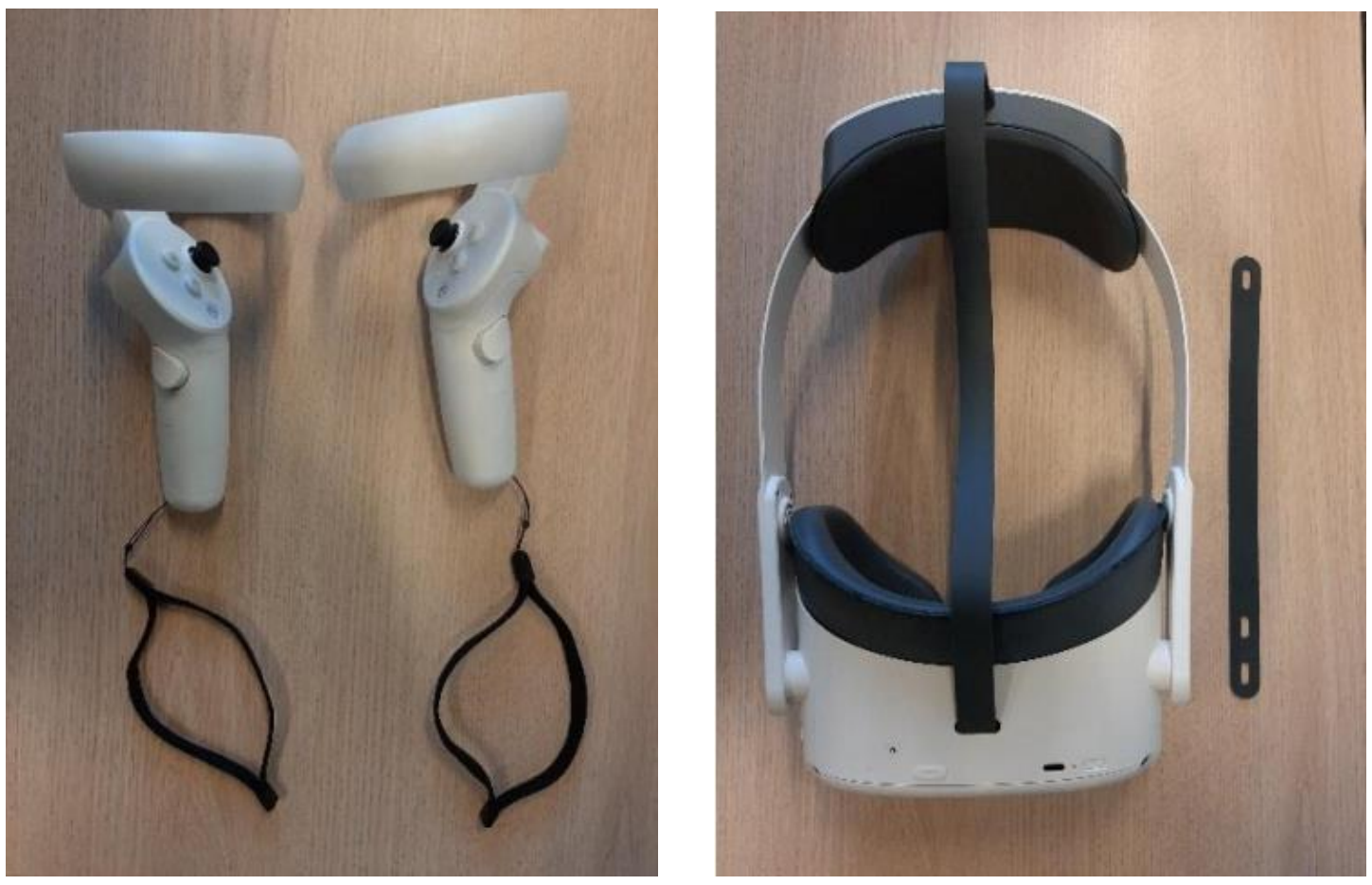

Image 1: The components of the Pico neo 3 need to be placed in the UV box in this setup

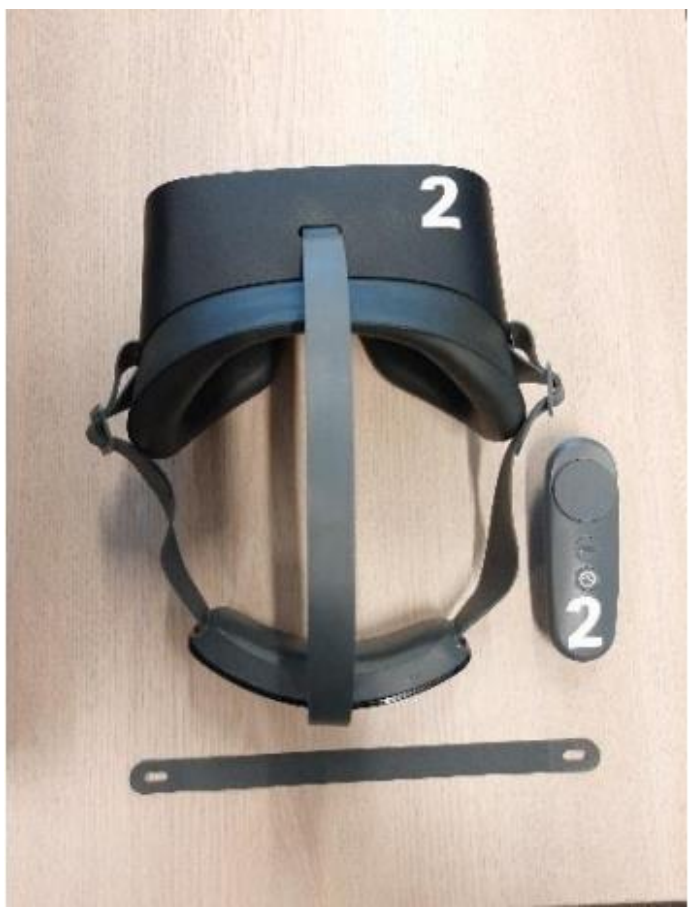

Image 2: The components of the Pico G2 need to be placed in the UV box in this setup

- Open the D25 en place the VR headset en the controller(s) in the D25.
- Position the components, according to image 1 and 2, to minimize shadowing.
- Close the D25.
- Disinfect the lidded storage box and your hands.
- Take the components out and store in the cleaned storage box.

Disinfect storage area

- Storage area reusable components
  The storage area is being cleaned once a month. This means that the available equipment is removed from the shelf and placed on a clean surface. Set up an organized system for separating clean and dirty components. The top and bottom of the shelf need to be cleaned with OxyFoam in combination with a disposable microfiber cloth, Bacillol 30 wipe of Oxy wipe S.

Using OxyFoam in combination with a disposable microfiber cloth:

For the disinfection of equipment, instruments, devices, and materials, and after contamination with blood and/or body fluids. It also has a cleaning effect in the absence of visible contamination. Suitable for daily disinfection, including for medical keyboards and mice.

Not suitable for oxidative surfaces like marble, copper like in (ear)thermometer or messing.

Not suitable for computer screens, touchscreens, (mobile) communication devices, non-medical keyboards and mice.

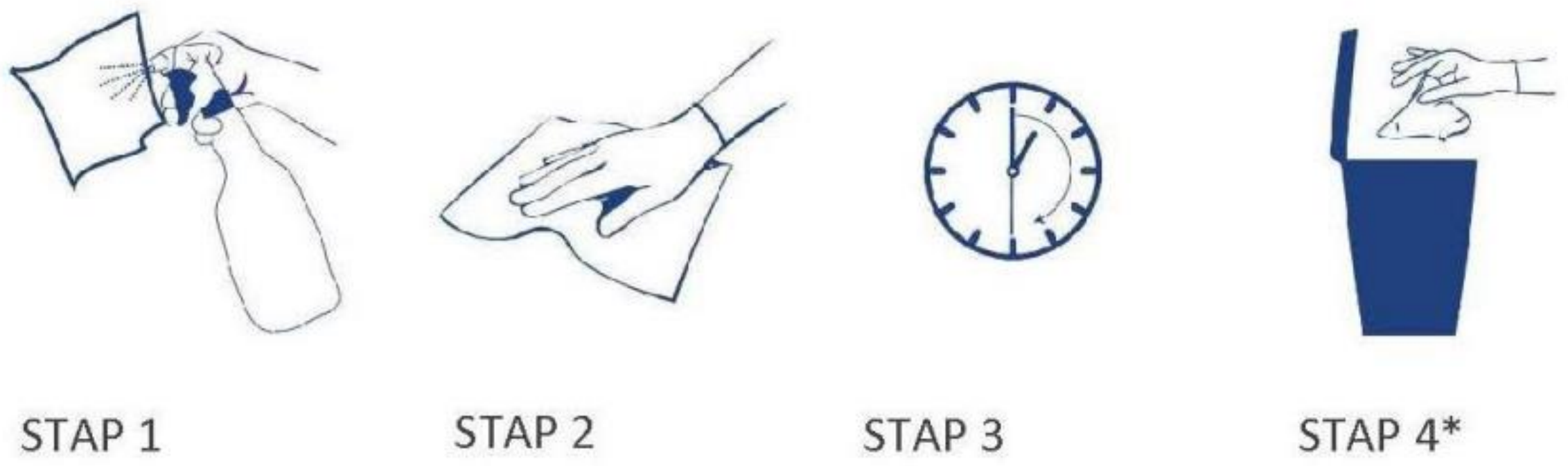

Step 1: Moisten the cloth generously (7 sprays per cloth); do not apply directly on surface!
Step 2: Apply on surface
Step 3: Wait until dry
Step 4: Throw away after use

Use Bacillol 30 wipe:

For the disinfection of small, smooth surfaces and non-critical medical devices, including (mobile) communication devices, touchscreens, and (ear)thermometers.

Use Oxy wipe S:

For the disinfection of small, smooth surfaces and medical devices used for patients in contact isolation due to confirmed Clostridium infection, or during an outbreak following consultation with the Hospital Infection Prevention (HIP) team.

Outside of a Clostridium isolation setting, this product is unsuitable for use on oxidizable surfaces—such as marble, copper (e.g., in ear thermometers), or messing—as well as on computer screens, touchscreens, (mobile) communication devices, and non-medical keyboards or mice.

Hygiene protocol VR headset

17-12-2021 versie 1.1

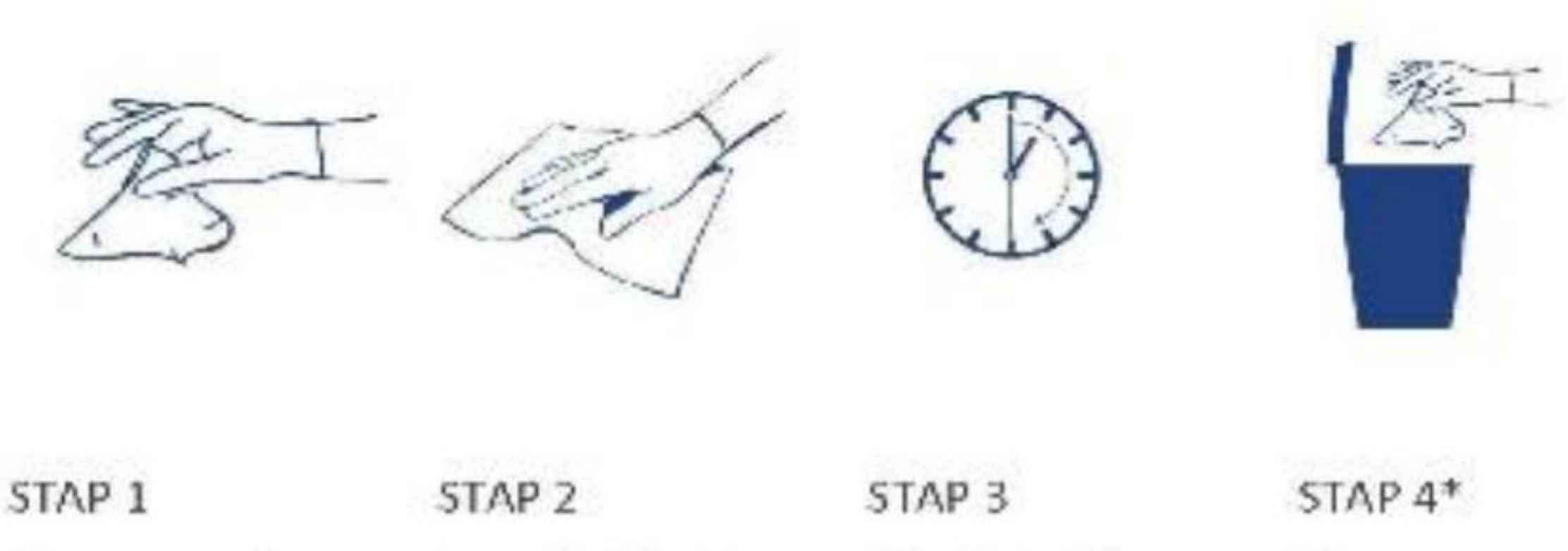

<u>Step 1</u>: Take a wipe from the package
<u>Step 2</u>: Apply on surface
<u>Step 3</u>: Wait until dry
<u>Step 4</u>: Throw away after use
